# Developing a Web-Based App to Assess Mental Health Difficulties in Secondary School Pupils: Qualitative User-Centered Design Study

**DOI:** 10.2196/30565

**Published:** 2022-01-10

**Authors:** Anne-Marie Burn, Tamsin J Ford, Jan Stochl, Peter B Jones, Jesus Perez, Joanna K Anderson

**Affiliations:** 1 Department of Psychiatry University of Cambridge Cambridge United Kingdom; 2 Peterborough National Health Services Foundation Trust Cambridgeshire United Kingdom; 3 Department of Kinanthropology Charles University Prague Czech Republic; 4 Institute of Biomedical Research of Salamanca Psychiatry Unit, Department of Medicine University of Salamanca Salamanca Spain

**Keywords:** mental health, assessment, young people, youth, schools, computerized adaptive testing, mobile apps, user-centered design, coproduction, qualitative study

## Abstract

**Background:**

Secondary schools are an ideal setting to identify young people experiencing mental health difficulties such as anxiety or depression. However, current methods of identification rely on cumbersome paper-based assessments, which are lengthy and time-consuming to complete and resource-intensive for schools to manage. Artemis-A is a prototype web app that uses computerized adaptive testing technology to shorten the length of the assessment and provides schools with a simple and feasible solution for mental health assessment.

**Objective:**

The objectives of this study are to coproduce the main components of the Artemis-A app with stakeholders to enhance the user interface, to carry out usability testing and finalize the interface design and functionality, and to explore the acceptability and feasibility of using Artemis-A in schools.

**Methods:**

This study involved 2 iterative design feedback cycles—an initial stakeholder consultation to inform the app design and user testing. Using a user-centered design approach, qualitative data were collected through focus groups and interviews with secondary school pupils, parents, school staff, and mental health professionals (N=48). All transcripts were thematically analyzed.

**Results:**

Initial stakeholder consultations provided feedback on preferences for the user interface design, school administration of the assessment, and outcome reporting. The findings informed the second iteration of the app design and development. The unmoderated usability assessment indicated that young people found the app easy to use and visually appealing. However, school staff suggested that additional features should be added to the school administration panel, which would provide them with more flexibility for data visualization. The analysis identified four themes relating to the implementation of the Artemis-A in schools, including the anticipated benefits and drawbacks of the app. Actionable suggestions for designing mental health assessment apps are also provided.

**Conclusions:**

Artemis-A is a potentially useful tool for secondary schools to assess the mental health of their pupils that requires minimal staff input and training. Future research will evaluate the feasibility and effectiveness of Artemis-A in a range of UK secondary schools.

## Introduction

### Background

Mental and substance use disorders are the leading causes of disability in children and young people worldwide [1]. In the United Kingdom, 1 in 7 secondary school pupils meets the diagnostic criteria for at least one mental health disorder [2]; however, <25% of young people with a diagnosable mental health condition are identified and offered support [2]. Mental health difficulties in adolescence predict a number of negative outcomes, including lower educational attainment, school dropout, substance abuse, delinquency, self-harm, and suicide [3]. Many mental health difficulties with onset in adolescence will persist into adulthood, negatively affecting an individual’s quality of life, productivity, and physical health [3] and resulting in high societal costs from increased health care use, unemployment, and criminal behaviors [4,5].

Schools have been championed as an optimal setting for the early identification of mental health difficulties in young people [6]. Young people identified in school settings are more likely to receive in school and specialist mental health support and have better long-term mental health outcomes compared with those identified in the community [7-10]. School-wide screening programs have been shown to be the most effective method for detecting mental health difficulties in young people compared with less systematic approaches [11]. However, schools are often reluctant to use this form of identification because of concerns regarding potential harms [11,12], adverse events [13,14], stigmatization of identified pupils [15], and increased demand for mental health services that exceeds available service provision [12,16]. There is also concern regarding the burden placed on schools, as universal methods of identification rely on cumbersome, costly, and time-consuming paper-based assessments, which teachers then need to score, interpret, and act upon [17,18].

The growing use of mobile technologies has accelerated the development and use of technology-based mental health interventions, particularly in younger age groups [19]. Studies focusing on young people’s perceptions of digital mental health interventions suggest that most youth feel comfortable using them [20], and almost 40% prefer web-based support to face-to-face therapy [21]. Web-based interventions are highly acceptable and viewed by young people as a way to avoid stigma associated with seeking and accessing help for mental health problems [21]. Some findings also indicate that digital mental health interventions reach young people who would otherwise delay help-seeking or not access support at all if face-to-face therapy were the only available option [20,22]. Research has identified a number of factors that predict young people’s engagement with digital mental health interventions. Level of detail and relevance of the content, acceptability, user-centeredness, personalization, and positive user experience (UX) contribute to continuous engagement [23,24], whereas concerns around privacy, validity, and credibility are likely to preclude young people from using digital mental health interventions [24,25]. These findings highlight the importance of involving end users in the design and development of mental health apps to maximize uptake, adherence, and effectiveness [23-25].

In recent years, technology-based mental health assessments using computerized adaptive testing (CAT) have gained traction because of their conciseness and accuracy and minimal burden on both patients and clinicians [26]. Adaptive tests for screening a range of mental health difficulties, including anxiety, depression, substance misuse, and suicidality, are now available in cloud-based environments and are being introduced in emergency departments, primary and secondary health care settings, student health clinics, child welfare, and justice systems [26]. Numerous simulation and evaluation studies have confirmed that CAT is a highly accurate method for assessing mental health difficulties in different populations [26-30]. However, very little is known about the feasibility and user perceptions of technology-based mental health assessments, particularly in the younger population.

In this study, we applied a user-centered design approach to develop Artemis-A, a web app that offers a rapid, practical, and feasible solution to mental health assessment in school settings. This paper describes the refinement of the app’s user interface (UI) and user testing to illustrate how coproduction is likely to improve functionality and ease of implementation.

### ARTEMIS: CAT Platform

The Artemis-A app for early identification of mental health difficulties in secondary school pupils was developed through an adaptation of an existing CAT platform (ARTEMIS) [28]. The application of CAT technology offers a personalized assessment by selecting each item based on the respondent’s answer to the preceding one [28]. CAT reduces the length of the assessment without compromising the accuracy of the results [26,28], enabling automated scoring and preparation of tailored reports [31]. In simulations [32], our testing in secondary schools and a real app pilot study [33] showed that users can complete the assessment in 7 questions (median 7, IQR 5-10) or 1-7 minutes depending on the age of the respondents.

The ARTEMIS platform incorporates a bank of 106 items derived from standardized psychological measures covering the most prevalent mental health difficulties. The measures are the Moods and Feelings Questionnaire [34], Revised Children’s Manifest Anxiety Scale [35], Leyton Obsessional Inventory [36], Rosenberg Self-Esteem Scale [37], Warwick-Edinburgh Mental Wellbeing Scale [38], and Schizotypal Personality Questionnaire [39], and there are an additional 8 items asking about symptoms of antisocial behaviors. For each assessment question, users choose a response (eg, *never*, *sometimes*, *mostly*, or *all the time*).

### This Study

The Artemis-A app is an adaptation of the root ARTEMIS platform. It was developed specifically for use in secondary schools to assess students’ mental health. As a screening tool, Artemis-A is intended for assessing the mental health of pupils with already identified mental health difficulties as well as those who have never had or currently do not have mental health concerns. We needed to design a UI that would be engaging for end users (particularly young people for whom web apps are an essential part of everyday life) and enhance the overall experience of those using the app across desktop and mobile platforms, as well as include a user-friendly administration panel for school staff to manage the assessments. We also wanted to explore whether the app would be acceptable in principle for stakeholders. The key objectives of this study are as follows: (1) to coproduce the main components of the app with young people and school staff to enhance the UI; (2) to carry out user testing, finalize the interface design, and explore the UX; and (3) to explore stakeholders’ views on acceptability and feasibility of web-based mental health assessments in schools.

## Methods

### Study Design and Setting

**Figure 1:**
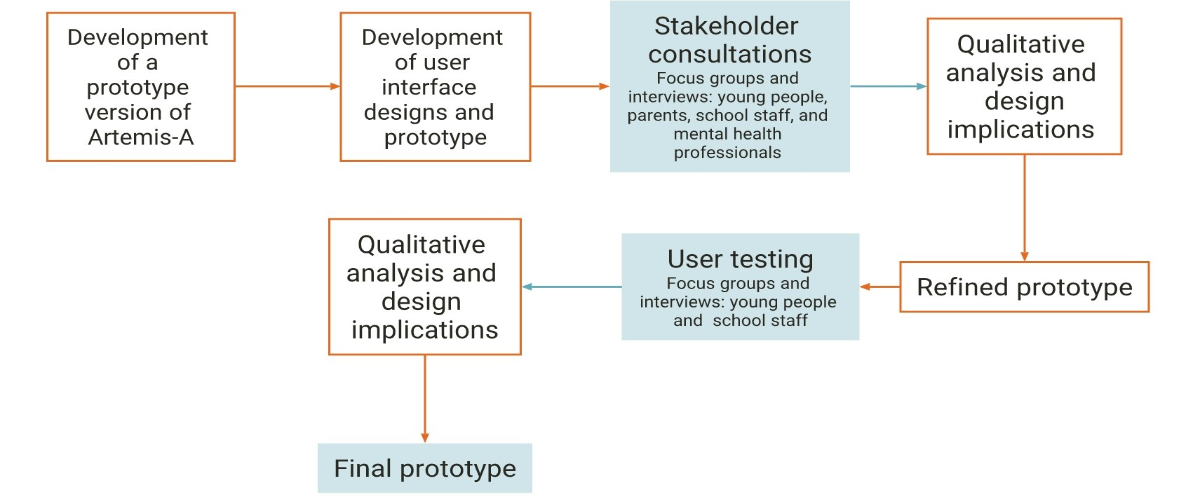
Coproduction process for Artemis-A.

The study took place from February 2020 to December 2020 and over 2 iterative design feedback cycles (Figure 1). We commissioned a digital design company to design and develop a high-fidelity prototype and a linked promotional website. The development of the app was underpinned by a user-centered design approach [40,41]. The initial coproduction work involved a stakeholder consultation with school pupils, parents, school staff, and mental health professionals to understand their needs and preferences. Feedback from the stakeholder consultation informed the design of the next iteration of the prototype. Unmoderated usability testing was conducted with pupils and school staff to gather feedback on the interface design and identify usability problems. In addition, participants’ views were sought regarding the use of web-based mental health assessments in schools.

### Ethics Approval

Ethical approval was granted by the University of Cambridge Department of Psychology Ethics Committee (ref: PRE.2019.081). Before the focus groups and interviews, signed consent was obtained from all participants (including parental consent for pupils aged <16 years).

### Participant Recruitment

We approached 4 secondary schools in the county of Cambridgeshire, United Kingdom to participate in the study. Key members of staff at each school assisted in the recruitment of pupils and parents and arranging the focus groups. Through existing networks, we purposively sampled school staff who were involved in mental health provision in their respective schools. In addition, we contacted mental health professionals who worked in local voluntary sector organizations specializing in children and young people’s mental health. Pupils were not selected based on their mental health, as we envisaged the app to be applied universally rather than on selected groups. Those who expressed an interest in taking part were contacted by a member of the research team and provided with an information sheet about the study. Participants were offered a £20 (US $26) web-based voucher as a thank you for their time.

For the stakeholder consultation, we recruited 32 participants, including 15 (47%) pupils (aged 11-15 years; 10/15, 67% female and 5/15, 33% male), 9 (28%) parents, and 8 (25%) school staff and mental health professionals. For the user testing iteration, we recruited 16 participants, which exceeded the recommended number for identifying 80%-85% of usability problems [42-44]. These 16 participants included 11 (69%) pupils (aged 11-15 years; 7/11, 64% female and 4/11, 36% male) and 5 (31%) school staff. Most pupils (across both iterations) attended a state-funded school (21/26, 81%), and 5 (5/26, 19%) attended a private fee-paying school. School staff had a range of roles, for example, school counselor, mental health lead, and director of welfare and inclusion. The mental health professionals worked in 3 voluntary sector organizations, and their roles were program director, a charity project worker, and a counselor who worked across schools.

### Procedures

For both design iterations, semistructured topic guides were developed for each stakeholder group. All participants completed a demographics form at the start of each session.

#### Iteration 1: Stakeholder Consultation Procedure

We held 5 focus groups: 2 sessions with pupils (7/15, 47% and 8/15, 53%, respectively), 2 sessions with parents (5/9, 56% and 4/9, 44%, respectively), and 1 session with school staff and mental health professionals combined (6/8, 75%). Groups took place on university and school premises and lasted between 1 and 2 hours. Key staff at participating schools assisted in arranging the time and location of the focus groups. In total, 25% (2/8) of school staff members were unable to attend the focus groups; therefore, we arranged to interview them individually over the phone. Each interview lasted approximately 45 minutes.

**Figure 2:**
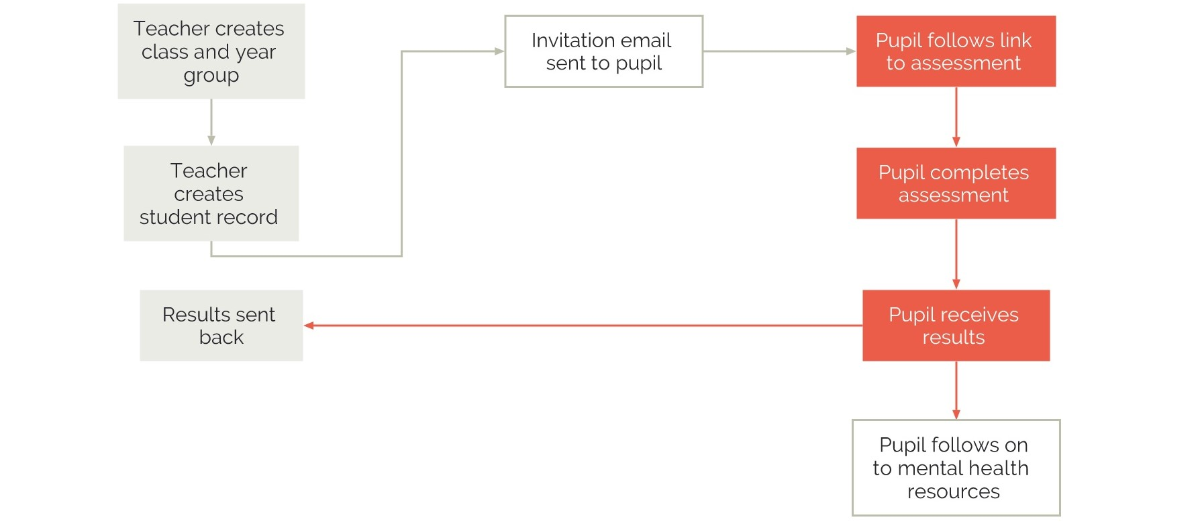
Artemis-A architecture and assessment workflow.

Members of the app development team attended the focus group sessions to collect feedback on the design and navigation preferences. At the start of each session, the participants were given a demonstration of the CAT platform to show how items were selected from the bank of measures. The presentation included an outline of the Artemis-A architecture and assessment flow (see the workflow map in Figure 2).

Following this, the app development team presented 3 visual mock-ups for both mobile and desktop platforms using an initial prototype and print assets. The 3 designs shared a visual identity to reflect the following overarching keywords—*positive*, *digital*, *modern*, *mental health orientated*, and *innovative*—but differed in terms of typeface, text information, and supporting graphical elements. The demonstration of the prototype gave participants a walkthrough of the various app pages based on different UI designs (ie, log-in, introduction page, assessment screen, feedback reports, and mental health resources). The participants were prompted to give feedback on the look and feel of the app, select the best design and functionality features, and suggest additional improvements. The participants were also asked about their general views regarding web-based mental health assessments in schools. User feedback was incorporated into the next version of the prototype and piloted by the members of the research team.

#### Iteration 2: User Testing Procedure

Feedback gathered during the stakeholder consultation was incorporated into the new version of the Artemis-A prototype, and a linked website was developed [45]. User testing took place in December 2020 and because of the UK COVID-19 pandemic restrictions at the time, these sessions were held remotely. School staff and pupils took part in an unmoderated usability assessment over a 2-week period. We asked pupils to complete the assessment several times at a time and place where they felt comfortable and to explore the functionality and take note of their UI and navigation experience. We also asked the pupils to check the privacy policy to see if they found the wording clear or confusing. School staff members were asked to complete several tasks, including using the administration panel to set up assessment groups, adding pupil details, sending invitation emails to participating pupils, and reviewing school outcome reports. We conducted 6 web-based feedback sessions—2 with staff (2/5, 40% and 3/5, 60%, respectively) and 4 with pupils (5/11, 46%; 3/11, 27%; 2/11, 18%; and 1/11, 9%, respectively). During the web-based sessions, we demonstrated the app using a videoconferencing software and a retrospective think-aloud technique and asked the participants to comment on the graphic design, usability, and navigability of the app and suggest improvements. Similar to the previous iteration, the participants were asked about their general views on implementing web-based mental health assessments in schools.

### Data Analysis

Audio recordings of the focus groups and interviews were transcribed, anonymized, and entered into NVivo version 12 (QSR International). The approach to analysis was both inductive and deductive. Initially, 2 team members (A-MB and JKA) independently read and coded a subset of the transcripts and then met to compare coding and discuss discrepancies. Both team members are experienced researchers in children and young people’s mental health. An initial coding framework was developed inductively from the data and deductively from the study research questions [46], including human factor elements (eg, design, content, and ease of use) [47]. All transcripts were then coded by A-MB, and 50% were coded by JKA. Throughout the analysis stage, both researchers met regularly to discuss and compare the coding, and the findings were discussed with the wider team. The researchers analyzed and interpreted the data and finalized the themes.

## Results

### User Preferences Identified Through Stakeholder Consultation

This section reports the results from the initial consultation with stakeholders. As young people would be the principal users of the web-based assessment, their choices about the UI design were prioritized over staff preferences. Example quotes from participants are provided in the text.

#### Preference for Simple, Minimal, and Consistent UI Design

**Figure 3:**
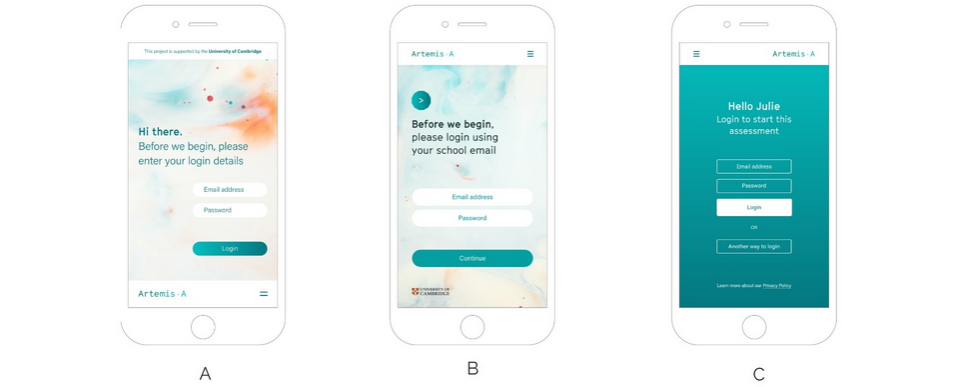
Schematic representations of the log-in page.

The participants were presented with three schematic representations via an initial prototype (see examples of the log-in page in Figure 3) and provided feedback regarding components of the UI, including the burger menu, logo, color scheme, and typography.

Most young people preferred option B because the design and color scheme were “calming” and “relaxing,” and they favored the large, bolder font as it was easier to read. It was important to young people that the design projected a professional feel because mental health was viewed as a serious matter. The inclusion of the University of Cambridge logo was liked because it carried weight and was seen as a trusted organization. Staff suggested adding the school’s logo to provide reassurance to pupils and parents.

The participants found the Artemis-A logo confusing because the arrow-shaped design suggested it was a clickable icon and would move the user to the next page (which it did not). This highlighted the need to redesign the logo:

It looks like it’s the going to do something-it’s an arrow, people will click on an arrow.

Young person 4, focus group 2

The 3-line burger menu was useful for orientation on the landing and introductory pages, but the consensus was that it should be positioned at the top of the UI to match convention and ease of access:

I think the menu should be at the top; they’re at the top on most websites, so most people would instinctively go to the top.

Young person 3, focus group 3

The participants thought the UI design should be as simple as possible, as other features may distract the user when completing the assessment. It was agreed that the burger menu was superfluous once the user started the assessment and could temporarily disappear while the users answered the questions.

The introductory pages on each schematic representation provided some background information describing what the assessment involved and what would happen to their results. Both young people and school staff said that the language used should be friendly and informal in tone and liked the personalization of option C, which included the pupil’s name on the log-in screen. However, several young people said that using the term *test* may cause young people to feel anxious and suggested *mental health assessment* or *quiz* as alternatives. Most participants preferred the text information presented in options B and C because it was clear, concise, and broken down into *bite-sized chunks*, which they found easier to read and digest. The vertical scroll felt more fluid to the participants for navigation rather than moving from page to page.

#### Young People’s Preference for Navigating the Assessment

**Figure 4:**
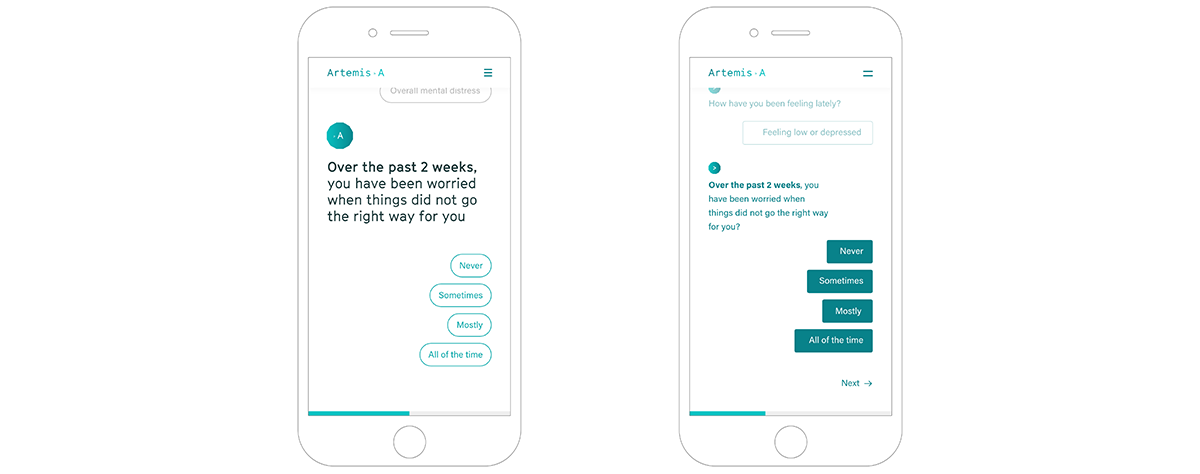
Options to navigate the assessment with and without a “next” command.

Young people were given a walkthrough of the assessment to demonstrate two potential navigation methods. The first method moved the users seamlessly to the next question once they selected their response. Young people preferred the second method, whereby users selected a response to the assessment question and then needed to validate their response before moving on to the next item. This 2-step process would provide the opportunity to pause and provide an error recovery before moving to the next question (Figure 4). This feature was particularly important to the younger participants who were concerned about accidentally pressing the wrong answer and biasing their results:

I’d press next ‘cause if you accidentally click a button and you just can’t go back then you might have to start it all over again.

Young person 1, focus group 1

#### Staff Requirements for School Administration of the Assessments

Using a workflow diagram as a guide (Figure 2), we asked staff what was the most feasible and secure way to distribute the assessment to young people and how they would like to administer the assessment within their schools. There was a clear consensus among staff that sending a link to individual pupils via the school email system would be the most secure method. They recommended that the link should be time-sensitive and remain active for up to 2 weeks.

Multilevel access to the administration panel was not seen as an important feature. The general view was that only a small team of school staff would need administration rights to distribute the assessment and access the results:

I would be tempted to keep that very closed in my school to a very selected group that have full access.

Staff 3, focus group 1

However, some staff requested that flexibility be built into the administration panel, which would allow for bespoke-level customization to create specific group distribution emails. For example, some requested the option to set up automatic emails so that the assessment could be sent at regular time points or to be able to resend the assessment to select groups of pupils on specific dates.

**Figure 5:**
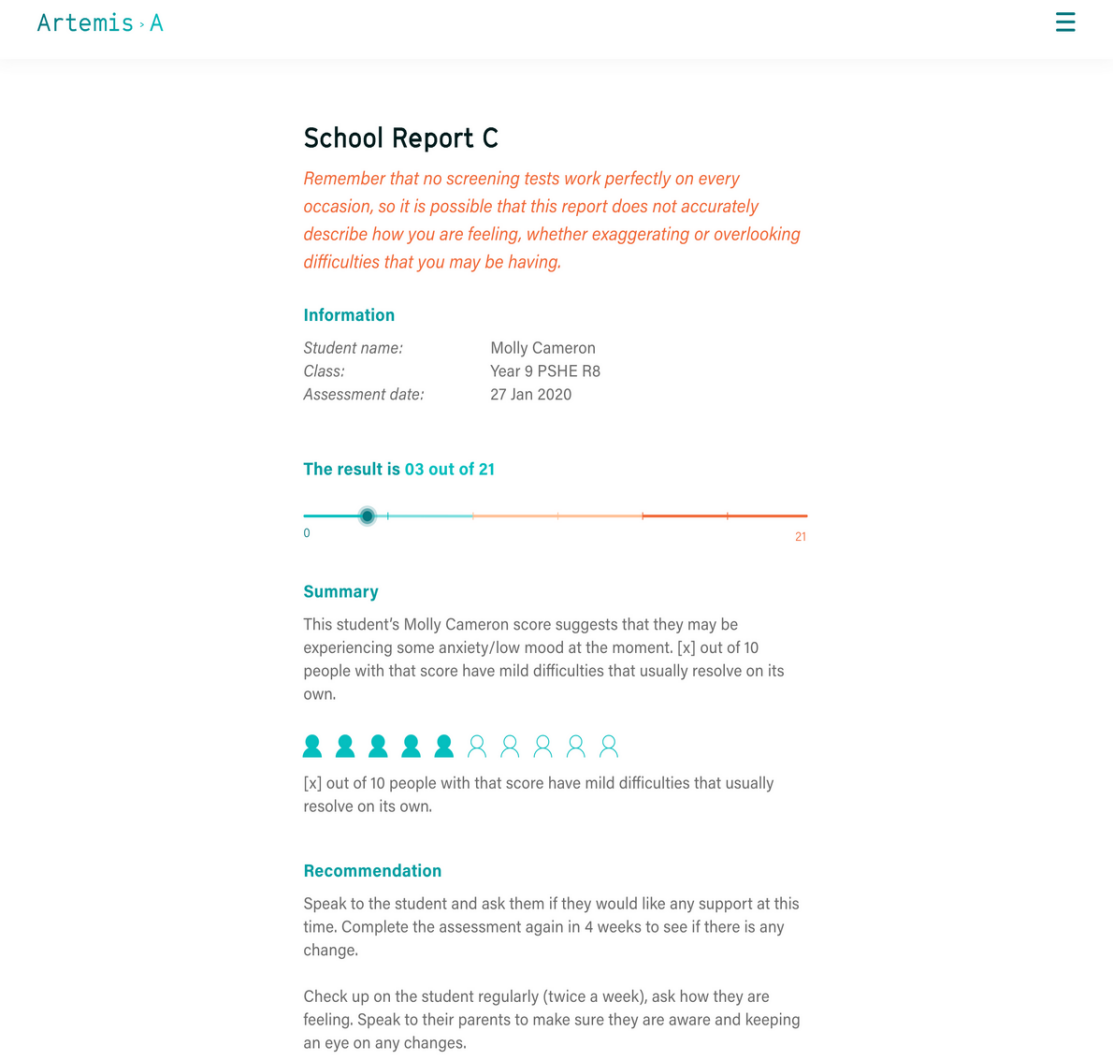
An example of a school report.

In total, 3 exemplar school reports with differing designs and layouts were presented to staff and mental health professionals. High scores in the report were highlighted in red and listed at the top of the report page to indicate a cause for concern (Figure 5). Staff particularly liked that the report flagged pupils who were at risk. However, they requested more features for data visualization of pupils’ results organized by school, year, class, or time. The use of color to convey the results was seen as particularly useful for school staff who may have low levels of mental health knowledge when interpreting the results:

I quite like the traffic light system because for people who aren’t trained it’s quite a simple way of seeing.

Mental health professional 1, focus group 1

#### Reports for Young People Need to Be Visual and User-friendly

Receiving an immediate assessment result was very important to young people. However, they suggested a number of features to make the report more user-friendly (eg, adding resources and helplines to the report page for ease of access).

Both staff and young people felt strongly that a visual representation of the result with a supporting explanation and recommendation would help young people understand the implications of their results better than a solitary numerical score:

I like the traffic light idea, I think young people are quite visual, but then I think we also need to be mindful that if it is red, they then need to know what the next steps are they can take within school as well as out of school.

Staff 6, interview 1

School staff were generally in favor of a traffic light method to convey results to pupils, but some were concerned that, if pupils saw their result falling in the red zone of the bar, this may increase their anxiety and distress. They suggested removing the visual presentation in high-scoring red zone reports. Importantly, reports should include supplementary advice and resources for young people and their parents, and pupils need to be informed about support from a member of staff if the assessment result is high:

I think there’s a lot of scope to personalise this for each school so you could even have the name of a member of staff in there, say, Mr Smith will be in touch with you.

Staff 8, interview 2

#### Schools Need to Customize the Mental Health Resource Pages

School staff requested bespoke-customization regarding the resource and support pages to tailor them specifically to the school population and to link to the school website. Although it was important to include national and local helplines, they wanted a section specifically for parents that provided information and resources on how to support their child. Staff suggested that a website should accompany the app, providing background information about the project and app development.

A total of three options for a resource page were presented to the young people. Young people preferred concise paragraphs where the text was broken up with subheadings because it was easier to navigate:

I like the paragraphs because they’re separated with the headers on option B.

Young person 8, focus group 2

### Findings From the User Testing

The results from the previous stakeholder consultations informed further design of the app. The prototype was tested over a 2-week period (11/16, 69% pupils and 5/16, 31% school staff). Below is the feedback from the user testing. Quotes are integrated into the text.

#### School Administration Panel Was Easy to Navigate but Lacked Flexibility

**Figure 6:**
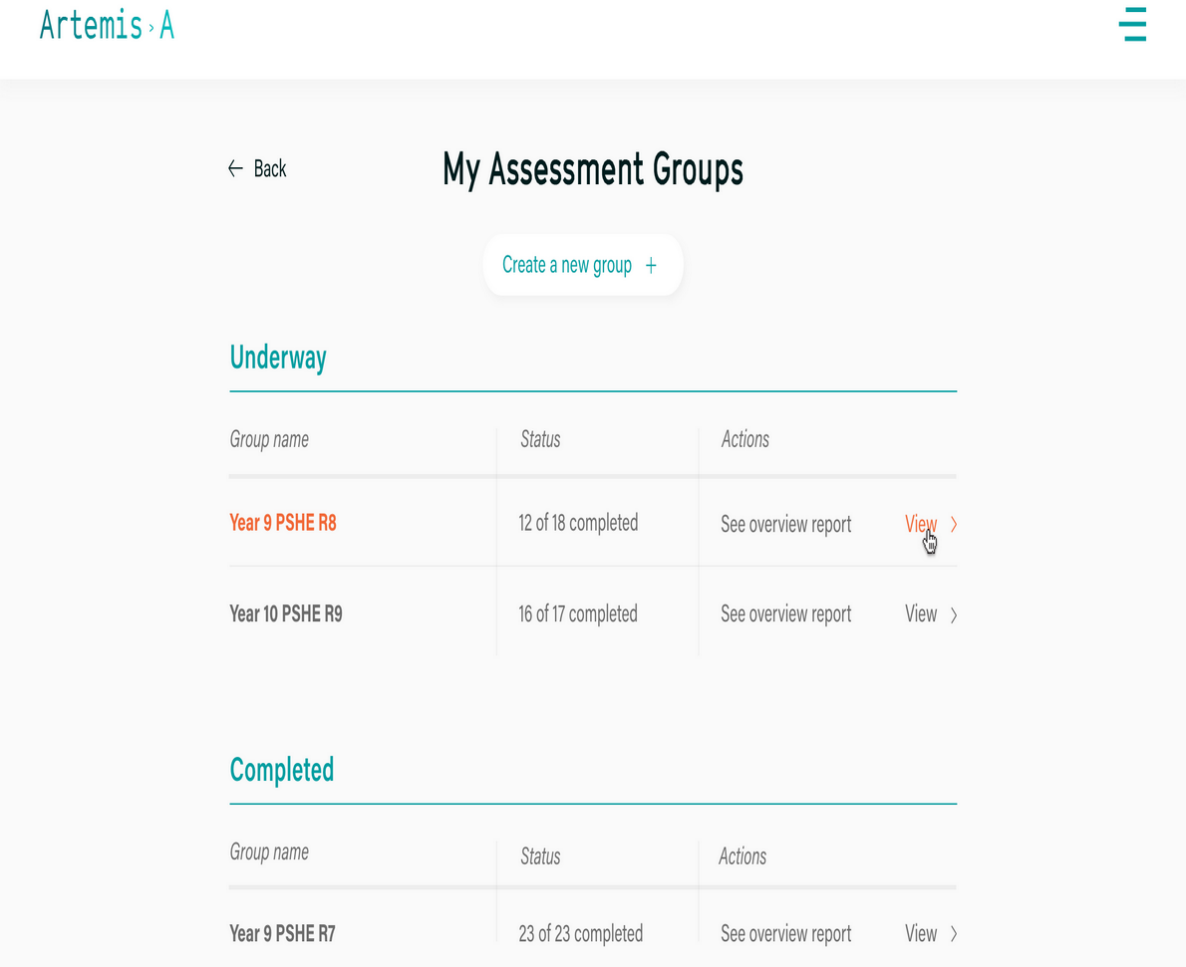
Administration panel for school staff.

To test the administration panel, staff members were asked to complete a number of tasks, including setting up student groups, adding individual pupil profiles, sending out invitation emails to the participating students, and generating group or individual reports (Figure 6).

Overall, the school staff found the administration panel easy to navigate; however, they reported some issues with finding particular functions. Setting up assessment groups and adding pupils was generally easy; however, staff preferred to add pupils’ details using a comma-separated values data file rather than manually uploading pupil information, and the upload function was difficult to find in the app. The staff members wanted a way of searching for an individual without needing to scroll through the list of all pupils; as they pointed out, in some schools, it may include >1000 names. In addition, they wanted more functionality to merge all year groups together to create the whole school report or separate year groups into subgroups (classes). Sending out invitations was generally easy to do at the “push of a button,” but they found that the emails went into the recipients’ spam folders. This involved staff needing to resend the invites and notify the pupils:

I think it’s fairly intuitive. I mean, it’s not an option-rich programme, is it, so it’s not like you’re getting lost in kind of different burger menus here, there and everywhere, it seems fairly straightforward to do it.

Staff 2, user testing dyad

Staff reported that the administration panel lacked flexibility in terms of selecting groups of pupils when sending the email invitation. Although these options existed, respondents pointed out that they could not find an option to select specific pupils (eg, those who were currently on the well-being team’s radar or receiving an intervention) to only send invites to. They asked that additional functionality be provided so that they could select a few pupils at a time or send invitations to all at once as school staff do not have the capacity to send separate emails:

What I think I struggled with the assessment group was, even if I were to put in a year eleven, we can only filter by one of those three things; I’d like to be able to just pinpoint, like, three students.

Staff 1, user testing triad

Reporting the results turned out to be the most challenging aspect of the administration panel. Many respondents reported having problems creating group reports, and those that did create them did not find them particularly informative. There was confusion regarding how to generate a pupil report and an overview report, and what the difference was between the two. Staff expressed the need for an administration panel feature that would enable them to visually present pupil data in different configurations; that is, for the whole school, year group, or class, as well as generate reports only for a certain period to be able to track fluctuations in pupils’ mental health (eg, before and after exams and every school term or semester). They requested some features for the individual pupil reports to enable them to track changes in pupils’ mental health, particularly if there were some concerns or if a pupil had received extra support or an intervention to see if it led to improvements. They also wanted to be able to see how an individual compared with other pupils in their class or year group and to have an option to access all individual reports for each pupil, not only the most recent one. Moreover, they wanted to know how long a pupil took to complete an assessment, as they were concerned that those who finished it very quickly might have selected responses randomly. It would be helpful if these pupils were flagged in the administration panel alongside those with extremely low scores so they could ensure that they answered truthfully. Finally, respondents agreed that it would be helpful if flags following the traffic light system were displayed next to each pupil’s name rather than only having red flags for pupils at risk:

If it could have little flags like it does on the other report, and colour coded so it shows who’s fine, so just at a glance you can see.

Staff 3, user testing triad

#### Young People Valued the App Design and Ease of Use

All young people completed the assessment several times during the testing period. Most pupils chose to complete it at home, citing privacy and lack of distractions as the main reasons for their choice; 1 young person completed the assessment on their bus journey home from school. Pupils generally found the app easy and simple to use and had a clear understanding of how it worked. As with the stakeholder consultation, several young people described the app as relaxing. Being provided with an immediate result was reassuring for pupils, and 1 described it as a huge “weight off their shoulders” (Young person 2, user testing 2).

Of the 11 pupils, 6 (55%) tested the app on both mobile and desktop platforms, 3 (27%) tested it on a desktop computer, and 2 (18%) tested it on a mobile device. For those who tried both, there was a strong preference for using the app on a desktop computer rather than on a mobile phone. A young person said it was easier to select a wrong response when completing the assessment on a phone.

Overall, young people liked the simplicity of the design and found the app easy to navigate. They liked the color scheme, although 1 said they thought it looked slightly clinical, and 1 suggested that there could be an option to personalize the color scheme. Several pupils asked for a progress bar to be added even though there was a progress bar at the bottom of the screen, which clearly indicated that it was not prominent on the interface.

#### Young People Valued Simple, Clear, and Informative Text Content

Young people liked that the assessment was called a mental health quiz rather than a test. They found the text information on the introductory pages easy to understand and said that the tone of the language used was calm and not forceful. They suggested changing *mental health problems* to *mental well-being* as it sounded more positive and encompassed the range of difficulties experienced by pupils. On the basis of the previous feedback, information was presented in smaller units that the participants found acceptable:

I think it’s quite good that you’ve sectioned it into paragraphs. I think if it was a big chunk of text then it would be quite overwhelming.

Young person 5, user testing 3

Most young people found the assessment questions easy to understand, although 1 young person suggested adding a box to say “I don’t understand the question” as a response option.

We provided a simple summary using age-appropriate language to help pupils understand the privacy and confidentiality of their data. The lay summary was intended to be used in addition to a comprehensive legal privacy policy. Pupils found the summary to be clear, and they felt reassured that *no other teachers will be able to see your results*. This statement was particularly important to some pupils, and they asked to have this as a stand-alone sentence so it would be more prominent and repeated separately on the introductory pages of the app. They said it would be useful if their school could include the name of the staff member who would manage the assessment and have access to the information.

#### School Reports Were Easy to Understand but Needed More Information

**Figure 7:**
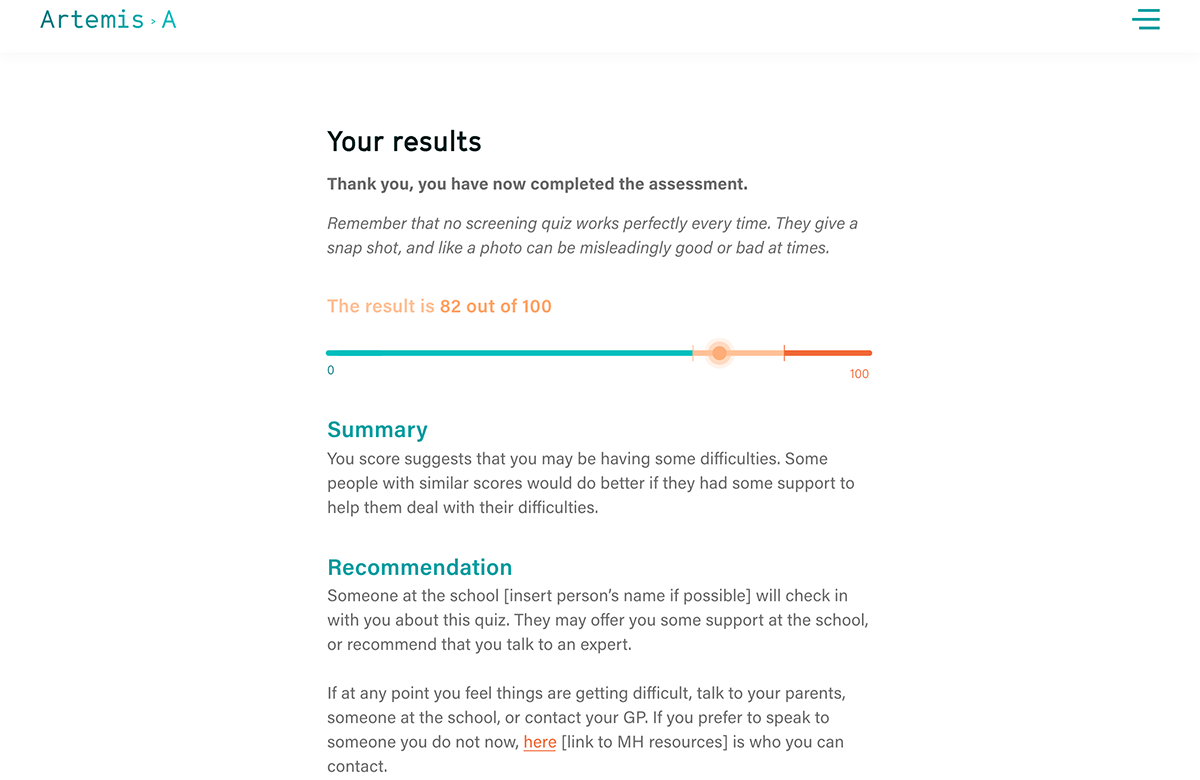
Example of pupil report with visual representation of score.

Most pupils received a report indicating a low to medium score (Figure 7). Following the suggestion made by the school staff in the first iteration, we removed the visual presentation of the results from the high score reports. However, during the user testing, pupils discussed their results with each other, and those who did not see a bar found it quite concerning. Those with a high score said they would still like the graphical presentation of their results. Pupils suggested that, instead of having a red zone on the graph for the highest score, having a green gradient going into gradually darkening amber would be less alarming. Young people also found the 0 and 100 marks on the graph unclear and suggested replacing them with text (eg, 0 replaced with “you are doing well” and 100 replaced with “you might be having some difficulties at this time”).

In addition, pupils wanted a more detailed explanation of what their particular score meant and, if it was high, more reassurance and suggestions about what they could do to improve their mental well-being. Finally, some young people suggested including the link to mental health resources on the results page together with a 1-button access to crisis support. School staff echoed the points made by the pupils, saying that the report should start with reassurance and that individual scores required more explanation.

#### Mental Health Resources Need to Be Tailored

Pupils suggested including local organizations (eg, charities providing mental health support) to give young people the option of having face-to-face contact with mental health practitioners and young people who were experiencing similar difficulties (eg, through attending group therapy sessions), as well as including information and support tools concerning how to cope at that moment (eg, cognitive behavioral therapy techniques and mindfulness). Links to websites should go directly to the web-based or counseling pages rather than the general home page. Staff wanted to be able to customize the list of mental health resources to include support offered within the school and add contact details of staff members who are involved in school mental health provision. A young person suggested expanding the list to include organizations providing support to lesbian, gay, bisexual, transgender, and queer (LGBTQ+) youth, and 1 suggested including the organizations’ logos as that would make them more recognizable.

### Themes on Acceptability and Feasibility of Web-Based Mental Health Assessments in Schools

Analysis of transcripts across both design iterations identified four themes relating to participants’ views regarding the acceptability and feasibility of the Artemis-A app: (1) schools as appropriate settings for web-based mental health assessments, (2) anticipated benefits of the app, (3) anticipated drawbacks of the app, and (4) suggestions for implementation.

#### Schools as Appropriate Settings for Web-Based Mental Health Assessments

Most parents and professionals viewed schools as an appropriate setting to assess young people’s mental health and for connecting identified pupils to local care and support. Professionals emphasized the value of third sector service provision integrating with schools.

Parents perceived high levels of self-harm within their children’s schools, particularly among the younger age groups and hoped that mental health assessments would identify those pupils in need. They valued the role that staff play in fostering good relationships with pupils, spotting those in distress, and providing good pastoral care. These relationships were seen as crucial for those pupils with difficult home lives (eg, those experiencing domestic abuse). Schools were described as stable and structured environments where vulnerable young people feel safe to seek help:

A lot of these young people find that schools are the most structured part of the day, the most stable part of their day.

Mental health professional 3, focus group 1

#### Anticipated Benefits of the App

Young people said that regular mental health checkups would give them reassurance and “peace of mind.” Parents echoed this and valued knowing how their children were doing. They hoped that, over time, regular web-based assessments would help normalize conversations about mental health and reduce stigma. An important finding was that young people said the app would provide a useful means for them to communicate their distress to adults without having embarrassing and awkward conversations with staff or parents:

I feel like this is a less scary way of talking about your mental health and letting people know than having to go face to face with someone.

Young person 2, user testing 3

Professionals thought Artemis-A was a useful and feasible tool that would provide schools with a quick, simple, and reliable method for mental health identification. They anticipated that it would be easy to administer and fit well with existing school structures and processes:

I think this is where it’s really exciting, the fact that you’re drawing lots of different standardized tests to get there, and the number of questions that they’re being asked is really minimal rather than going through a whole questionnaire.

Mental health professional 1, focus group 1

Professionals and parents discussed how the app could be used for systematic universal screening, where all pupils are assessed and monitored over time. They noted that universal screening would avoid some pupils feeling singled out and harms associated with stigma. Moreover, the app would capture young people who may be struggling with their mental health and may otherwise go unnoticed:

This app then may kind of sweep up those that aren’t maybe showing major signs of issues.

Mental health professional 2, focus group 1

#### Anticipated Drawbacks of the App

The potential adverse effects of using Artemis-A in schools were raised during both parts of the study. Parents voiced concerns that using the app may exacerbate young people’s anxiety about their mental health or lead to bullying, stigma, and feelings of shame:

When they’re filling in the app they might be ashamed of it.

Parent 3, focus group 4

Some participants thought screening all pupils with the app would “open a can of worms” by identifying large numbers of pupils which in turn could potentially overwhelm schools. The long waiting times for specialist services could leave schools needing to fill the gap in provision, but staff may not be equipped with the skills or training to do this.

A consistent theme was identified across both study iterations, which showed a high level of concern among young people regarding their privacy and the confidentiality of their data. Young people wanted privacy when completing the assessment and did not want their friends to know about their problems. This influenced their preference for completing the assessment at home away from others, rather than at school:

I would much prefer to do it at home because I’d feel a lot safer.

Young person 3, user testing 5

Young people were also concerned about the confidentiality of their data within the school and clearly articulated that they would want to know which staff members would have access to their results. They suggested that there should be a designated staff member administering and managing the assessment so they would be aware of who they could speak to about their results:

You might not want them to know about it, you might just want to keep it private.

Young person 1, user testing 4

#### Suggestions for Implementation

During discussions, the participants made clear suggestions for implementing Artemis-A in schools. Staff felt that Artemis-A should be managed by a small team of trained staff to ensure that pupils’ confidential data would not be accessible by the wider staff. Comparisons were made with existing school safeguarding systems, which have restricted access. It was seen to be important that young people are given a sense of agency about where and when they complete the assessment and that they feel in control of what happens to their data. It was suggested that pupils be provided with information explaining that their data are private and protected and be made aware of the designated members of staff who will have access to their data. Moreover, staff should have conversations with pupils before sharing their information with others (eg, parents or form tutors).

Across all participant groups, there was a strong view that parental involvement should be kept to a minimum, unless young people were at crisis points and then safeguarding protocols would be followed. Parental consent (opt-in) could be obtained when pupils join the school or at the beginning of each academic year as this would not require significant resources.

Staff and mental health professionals expected that schools would use Artemis-A for both universal and selective mental health screening, but schools should be made aware of the benefits and drawbacks of each approach. Although Artemis-A was perceived to be a useful tool for mental health assessment, the participants noted the lack of specialist support available for those identified. They felt strongly that schools should include mental health education in the curriculum, training for staff to increase their knowledge and skills regarding mental health, and improved accessibility to effective interventions:

So I think is this fantastic but it has to be a part of something bigger.

Mental health professional 2, focus group 1

## Discussion

### Principal Findings

This study describes the coproduction work with school pupils, parents, school staff, and mental health professionals to ensure their needs and preferences were incorporated into the app design. The participants were enthusiastic to be part of the design process and had clear views regarding the design and how it should work. Incorporating the views of multiple stakeholders increases the acceptability and feasibility of the app and also boosts uptake and retention [40,48]. A number of suggestions made by the users are likely to improve the overall functionality of the app as well as facilitate its implementation and adoption in schools.

Initial stakeholder consultations with pupils and school staff directed the UI design, including color scheme, typography, text content, graphical elements, and preferred navigation features. Pupils specified a minimalist design, a personalized UI, and user-friendly text content presented in small units with clear subheadings. They favored a 2-step process to minimize user error when selecting an assessment response and the removal of the burger menu during the assessment process. School staff and mental health professionals provided valuable insights relating to the features for assessment distribution, administration, and outcome reporting for schools. They highlighted the need for schools to customize the administration panel and mental health resource pages. Although the outcome reports were liked and easy to understand, the staff expressed concerns about reports increasing distress in high-scoring pupils.

In subsequent user testing, staff found it easy to add individual profiles, set up groups, and send out invitation emails to pupils. However, staff reported that some features of the administration panel were not intuitive and that there was a lack of functionality for generating group or individual reports. They required more flexibility for searching through pupil records and for data visualization to track fluctuations in pupils’ mental health by cohort at particular times of the year (eg, before and during exams). An important design change would be to have flags next to the names of at-risk pupils and move these pupils to the top of the list.

During user testing, young people liked the design of the UI and found the text content easy to read when presented in smaller units with subheadings. The lay privacy summary was particularly useful to young people because it reassured them of their privacy and confidentiality. They found the app easy to navigate and comfortable to use, but they asked for the progress bar to be more prominent. They valued the resource page but suggested more local third sector organization support should also be included.

It is important to note that different stakeholders sometimes had conflicting expectations regarding the content of the app. Initially, school staff expressed a concern that providing a visual representation of the results to students who achieved high scores in the red zone (indicating significant mental health risks) could be distressing for young people. In response to this, we removed the graph from reports for students who achieved the highest scores; however, in the user testing feedback sessions, young people admitted that, although seeing their score being in the red zone may be worrying, they were more concerned about why the format of their feedback was different from that of their peers. They suggested replacing the red zone with an amber color gradient that darkens as the score increases. This is an important finding showing the importance of testing an intervention with different user groups as well as finding a balance when addressing conflicting expectations to ensure wider acceptability.

School staff viewed the app as useful, feasible, and congruent with existing school structures and processes. Pupils and parents considered it a useful tool to monitor young people’s mental health over time and ensure that those who may be struggling are identified. They were also convinced that regular mental health checkups would normalize conversations about mental health and help reduce stigma. However, pupils, parents, and staff all voiced concerns that using the app may exacerbate young people’s anxiety about mental health or instigate bullying, stigma, and feelings of shame. Pupils were also concerned about the confidentiality of their data and who within the school would be made aware of their results.

School staff thought the app would be relatively easy to implement in schools; however, they stressed the importance of young people being given a sense of agency about where and when they completed the assessment and being informed about what happens to their data, as well as the importance of the availability of resources to address the mental health needs of young people identified by the app.

### Comparison With Previous Work

Although CAT has previously been applied to facilitate mental health assessments in different patient groups [26,27,49,50], the use of CAT to assess children and young people’s mental health is relatively new [30]. In contrast, early prototyping, the involvement of users, and continuous iteration are key principles of a user-centered design approach [40,51], which in recent years has become more prominent in the development of mental health web apps [52-56]. Ospina-Pinillos et al [52,53] applied user-centered design methodologies with young people, their carers, and health professionals in Colombia [52] and Australia to culturally adapt a Spanish version of a web-based Mental Health eClinic. The design process involved four iterative phases: co-design workshops, knowledge translation, tailoring to cultural context, and one-on-one user-testing sessions. Similar to our findings, young people valued flexibility and personalization of the app and stressed the importance of privacy and data protection. The color palette users selected in co-design workshops was blue-green with orange, very similar to the preferences of our study participants [52]. Interestingly, in both countries, all young people agreed that assessment results should be displayed immediately, whereas in Colombia, some mental health professionals were concerned that the pertinence of the results could be a source of distress. Their proposed solution was to give users a choice of whether they wanted to view their results straight away or wait to review them with a professional present [52]. In Australia, young people liked the traffic light system (similar to the one proposed for our app) used in the reports [53]. In our study, school staff suggested the inclusion of measures of eating disorders and suicide risk, whereas in Colombia, practitioners and young people wanted to include measures of poverty level, exposure to violence, and posttraumatic stress disorder because of the specific context.

A review of existing mental health apps conducted by Bakker et al [57] resulted in a number of evidence-based recommendations for app development. The authors highlighted the importance of automated, seamless tailoring; the simplicity of the interface and ease of navigation; short sentences; and simple, concrete language. Our study identified key areas of improvement to the UI and UX that are consistent with the usability heuristics by Nielsen [58] and reflect the above recommendations. The findings from this study indicated that the interface needed a minimalist design without unnecessary elements that may distract users. Textual information needed to be presented in short paragraphs, which is consistent with the recommendations for developing web apps whereby textual information is chunked to support users’ capacity for working memory [59] and to account for slower readability on screens [60,61]. The 2-step process incorporated into the assessment aided in error prevention [62]. We found that the Artemis-A icon usability was poor and did not successfully communicate meaning [59]. Regarding content, Bakker et al [57] stressed the importance of including quick-access links to crisis support, something that was also highlighted by our study participants.

Our findings on the potential benefits and drawbacks associated with using the app in schools are in line with findings from other studies on mental health assessments in schools. Systematic, universal screening correctly identifies more at-risk pupils, including those with internalizing disorders who are often missed, than any other identification method currently used in schools [11]. Moreover, pupils identified in school are more likely to receive support from mental health services [7-9] and have better long-term mental health outcomes compared with those identified in community health care settings [63,64]. Studies examining the iatrogenic effects of mental health assessments in schools are scarce. To our knowledge, there are no studies focusing specifically on harms associated with general mental health assessments in schools; however, a recent systematic review and meta-analysis found no evidence of adverse events following screening for self-harm and suicide-related behaviors [65]. In our study, the participants thought the app would be relatively easy to implement in schools. School staff generally prefer mental health programs delivered on the web over those delivered face to face [66], mainly because of the flexibility and cost-effectiveness of web-based interventions [59]. Although there are no studies examining pupils’ views on web-based mental health assessments, evidence shows that young people are happy to engage with digital mental health programs and perceive them as less stigmatizing and more accessible [21,67,68].

### Study Limitations

Participants were recruited from a small number of schools in the East of England, and their views may not be representative of pupils and staff from other schools or regions. The app features and design preferences may not be applicable or practicable for other schools and staff members. To ensure a range of views, we included young people from different types of schools (ie, state-funded and privately funded). However, we acknowledge that our sample lacked diversity, and further work should ensure the participation of pupils from a broad range of backgrounds, including young people from marginalized groups such as LGBTQ+ and young people with disabilities. Therefore, the app design may not be acceptable or accessible to all pupils.

### Implications for Practice

The findings from our study suggest that using apps for the identification of mental health difficulties is a feasible and acceptable alternative to paper-based mental health assessments. The application of CAT technology had a positive impact on the willingness of both pupils and school staff to use the proposed app because of its rapidity and minimal user burden.

Our study highlighted the importance of involving the end users in the design process from the early stages. Young people who participated in the focus groups stressed the importance of particular app features, including privacy and data protection, instant feedback, and access to crisis support, which our team had not considered as vital. End users also made important suggestions about the language (ie, making it positive and unthreatening and presenting text in bite-sized chunks). Involving users and allowing them to take the lead during the design process ensured that the final product was highly acceptable to young people and that the assessment invoked positive feelings rather than anxiety about one’s mental health.

It is also important that participants are given a small number of design options to choose from rather than being asked to generate new ideas. We found that limiting options helped maintain the focus during group discussions, promoted a better use of time, and made participants’ suggestions easier to actualize.

Our study also highlights the importance of involving a range of stakeholders before implementing school-based interventions. The participants provided important information that will guide future implementation strategies (ie, how Artemis-A will be put into practice in schools). The participants identified a number of issues that could affect acceptability (eg, stigma, data confidentiality, and privacy) and feasibility (eg, designated staff to manage the app and access data). This information is crucial for promoting the successful implementation of web-based mental health assessments in school settings.

### Future Work

Future work will include item calibration and validation with a younger secondary school population, which will enable us to further refine the CAT algorithm. We will test the usability of Artemis-A with more diverse populations to ensure that it is acceptable to young people with a range of backgrounds and accessible by pupils with disabilities. Furthermore, we will evaluate the feasibility and effectiveness of Artemis-A in a range of UK secondary schools and explore barriers to and facilitators of implementation.

## Abbreviations

CAT: computerized adaptive testing
NIHR: National Institute for Health Research
UI: user interface
UX: user experience
